# Implications of Public Interest in Colonoscopy: Analysis of Google Trends Data From 12 European Countries

**DOI:** 10.7759/cureus.42395

**Published:** 2023-07-24

**Authors:** Tomasz Skrzypczak, Anna Skrzypczak, Małgorzata Skrzypczak

**Affiliations:** 1 Faculty of Medicine, Wroclaw Medical University, Wroclaw, POL; 2 Faculty of Dentistry, Wroclaw Medical University, Wroclaw, POL; 3 General Dentistry, Private Dental Practice, Krobia, POL

**Keywords:** european union, colorectal cancer, google trend, google trends healthcare, colonoscopy

## Abstract

Introduction: Colorectal cancer (CRC) is one of the deadliest diseases in the European Union. Colonoscopy remains the gold standard of CRC screening. Analysis of colonoscopy-related Google Trends (GT; Google LLC, Mountain View, California, United States) data could provide useful information regarding interest in colonoscopy and potential barriers making patients unwilling to attend screening programs.

Methods: Data were collected using GT for the main search term “colonoscopy” and the two most related queries. Colonoscopy volumes were extracted from the Eurostat database. Due to limited Eurostat data availability, analysis was performed from January 2004 to December 2015 for each of the 12 included countries.

Results: Univariate linear regression analysis demonstrated statistically significant correlations between annual search volumes of “colonoscopy” and the annual number of colonoscopies performed in included countries (*R^2^* = 0.923, *P*<.001). Trend analysis showed that the cumulative search volumes for “colonoscopy” gradually increased through the analyzed period. The spectrum of the most related queries encompassed “preparation for colonoscopy”, “endoscopy”, “after endoscopy”, “colon”, “colonoscopy diet”, “virtual colonoscopy”, “colonoscopy under anesthesia”, “waiting times for colonoscopy” and “colonoscopy price”. For eight out of nine queries, statistically significant correlations with procedure volumes were revealed.

Conclusions: GT could be a useful tool in assessing public interest in colonoscopy. Potential barriers that prevent people from attending CRC screening programs were identified. The study demonstrated that the internet has become an important field for CRC screening promotion. GT utility for colonoscopy and CRC screening providers was highlighted. This was the first analysis of GT data in colonoscopy focused on European countries.

## Introduction

Colorectal cancer (CRC) is the second deadliest cancer in the European Union (EU) [1]. Approximately 170,000 people are dying from CRC annually in the EU and this number is expected to rise [1,2]. Projected growth associated with the aging population is boosted by increasing incidence among individuals below 50 years of age [3]. The rising prevalence of obesity, lack of exercise, alcohol, processed meat, air pollution, and even urbanization contribute to an increase in CRC incidence [4,5]. CRC is both preventable and curable if detected early enough [1]. Unfortunately, most of the patients are detected at advanced stages, which is associated with unfavorable prognosis [1]. If the EU was able to diagnose more patients at early stages, up to 130,000 lives could be saved per year, and more than € 3 billion in healthcare budget savings could be guaranteed [1].

Colonoscopy is still considered the gold standard CRC screening method [6]. Most guidelines recommend screening average-risk individuals between ages 50 and 75 using a colonoscopy every 10 years [7]. Despite multiple programs implemented in the EU member states, only 14% of EU citizens aged 50-75 years participate in CRC formal population-based screening programs [1]. Many barriers were associated with low participation rates [1,7]. Health system issues, including inadequate access, high costs, and insufficient capacity to ensure screening colonoscopy for everyone who qualifies were identified as the most burdensome [8]. Proper capacity was indicated as one of the key aspects that require improvements [1]. Furthermore, patients’ matters such as fear, psychosocial factors as well as CRC awareness and understanding play a significant role in participation rates [1].

Google Trends (GT; Google LLC, Mountain View, California, United States) is a public Internet tool that allows the user to track how frequently a certain phrase is queried in the Google Search services [9]. Multiple studies identified the value of GT in cancer screening and assessing public health [10,11]. Recent investigations used GT to assess trends in patients’ interest in neurological, bariatric, and cataract surgery [12-14]. GT also was widely used in multiple studies related to colonoscopy [15-19]. It was utilized to investigate colonoscopy search volumes during the coronavirus disease 2019 (COVID-19) outbreak [15,16,19], associations between 10-year CRC mortality and GT [18], and the quality of the information provided by Google Search [17]. These studies were focused on English-speaking countries and most of them explored only search volumes related to the United States. No study to date examined GT as a potential tool for colonoscopy interest estimation in European countries with reliable statistical data. European countries should be targeted, due to a negligible number of GT studies dedicated to this region [12,20].

The study’s primary aim was to examine GT as a reliable tool for estimating colonoscopy public interest in European countries. Secondly, potential patients’ concerns encompassed in searched terms spectrum were evaluated.

## Materials and methods

GT allows for personalized queries based on phrases used, geographic location, time, result format, and search category. With these, GT generates databases and graphs with numbers representing search interest associated with the peak popularity of that term, which is given a value of 100 [12,21].

The search term “colonoscopy” was input into GT in the native language of each country [12]. Then, a list of the most related queries was generated and translated into English [12]. Each query was assessed for suitability. Two of the most yielded search queries, not leading to similar results, were included in the further analysis [12]. “Preparation for colonoscopy”, “endoscopy”, “after endoscopy”, “colon”, “colonoscopy diet”, “virtual colonoscopy”, “colonoscopy under anesthesia”, “waiting times for colonoscopy”, “colonoscopy price” were selected from all included countries. “Gastroscopy” and “sigmoidoscopy” represented different procedures, and thus were excluded from the study. “Endo clinic”, “Endoscope”, “Endo hospital” were excluded due to very low search yield (popularity peak < 10). Translations of the search terms were obtained with Google Translate.

Univariate linear regression analysis was conducted to estimate the correlation between the annual number of performed colonoscopies with or without biopsies, according to data from the Eurostat [22] and GT searched terms query volume per year. To investigate other possibilities for changes in the trends that potentially occurred, weighted median age and total population of included countries were utilized to explain changes in endoscopy volume, in accordance with Eurostat data [22]. The root of R2 was extracted to calculate correlation coefficients (r) [23]. These were classified as very strong 1.0$\geq$r$\geq$0.90; strong 0.89$\geq$r$\geq$0.70 and moderate 0.69$\geq$r$\geq$0.40 [24]. Due to a lack of colonoscopy volume records after 2015, the period from January 2004 to December 2015 was investigated. JASP version 0.17.1 (Released 2023; JASP Team, Amsterdam, The Netherlands) and Microsoft Excel version 16.59 (Microsoft Corporation, Redmond, Washington, United States) were utilized for all statistical analyses. P<0.05 was considered statistically significant.

Denmark, Germany, Ireland, Spain, France, Italy, Hungary, Portugal, Slovenia, Finland, Sweden, and the United Kingdom (UK) were included and met the following criteria: No more than one Eurostat report was missing, sufficient data was available in GT system to generate a list of the most related queries, and translation of “colonoscopy” was possible for each of the analyzed countries. 

## Results

**Figure 1:**
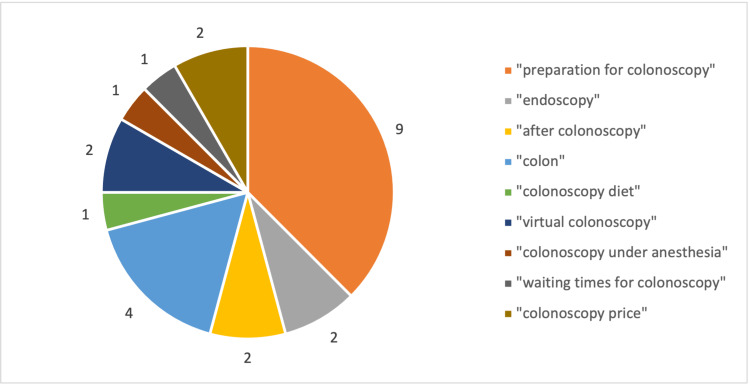
The most related queries to "colonoscopy" in 12 analyzed countries

The two most related queries to colonoscopy were investigated for each country. In the 12 included countries, 24 options were possible. The most related queries are presented in Figure 1.

The most prevalent term was “preparation for colonoscopy”, found in Denmark, Germany, Ireland, Spain, Italy, Portugal, Finland, Sweden, and the UK (9/12). Second, the most popular related query was “colon”, found in Ireland, Spain, France, and Hungary (4/12). “Endoscopy” was revealed in Denmark and Sweden (2/12), “after endoscopy” in Germany and UK (2/12), “virtual colonoscopy” in Italy and Portugal (2/12), and “colonoscopy price” was found in Slovenia and Finland (2/12). “Colonoscopy diet”, “colonoscopy under anesthesia”, and “waiting times for colonoscopy” were revealed in France, Hungary, and Slovenia, respectively.

**Figure 2:**
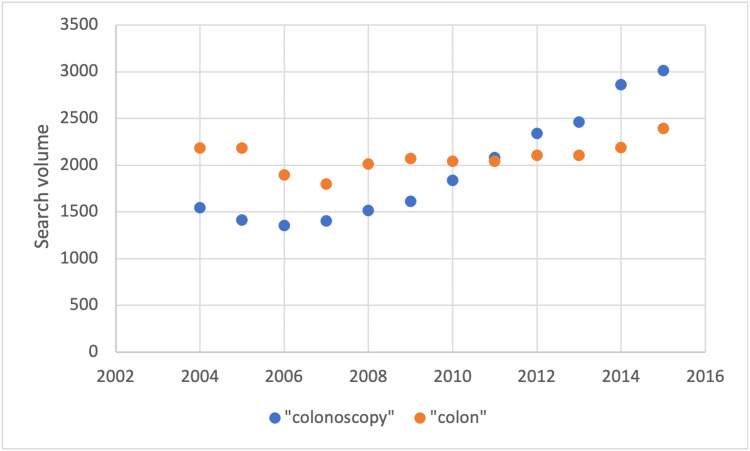
Trends in "colonoscopy" and "colon" search volumes from 2004 to 2015

“Colonoscopy” and “colon” were the most prevalent terms with mean 1954 ± 589 and 2087 ± 151 search volumes. From 2004 to 2015, there was a positive trend in the number of “colonoscopy” queries, while “colon” remained stable. These changes were presented in Figure 2. 

The most related queries “preparation for colonoscopy”, “endoscopy”, “colonoscopy price”, “virtual colonoscopy”, “colonoscopy under anesthesia”, “waiting times for colonoscopy”, “after colonoscopy” and “colonoscopy diet” were significantly less prevalent with 252 ± 194, 175 ± 78, 53 ± 74, 67 ± 21, 30 ± 34, 14 ± 18, 41 ± 28 and 8 ± 4 search volumes, respectively.

Univariate linear regression analysis over time from 2004 to 2015 revealed a statistically significant correlation between the annual volume of performed endoscopies in all included countries and the cumulative number of Google Search volumes for “colonoscopy” (R^2^ = 0.923, P<.001). Overall, the correlation between “colonoscopy” and the number of procedures was very strong (r=0.961). Apart from Denmark (r = 0.683), Hungary (r = 0.854), and Slovenia (r = 0.872), a very strong correlation (1.0 r 0.90) was observed for each of the included countries. All correlations were statistically significant. Detailed information related to each country is presented in Table 1.

**Table 1:** Results of univariate regression analysis between “colonoscopy” search volumes and the annual number of colonoscopies performed in each of 12 included countries P<.05 was considered statistically significant

Country	Searched term	Beta coefficient	Lower 95% CI	Upper 95% CI	P	r	R^2^
Denmark	koloskopi	478.041	117.950	838.132	0.014	0.683	0.467
Germany	darmspiegelung	2191.163	1702.24	2680.09	< .001	0.953	0.909
Ireland	colonoscopy	426.359	374.51	478.207	< .001	0.984	0.968
Spain	colonoscopia	2623.083	2263.76	2982.41	< .001	0.979	0.959
France	coloscopie	7902.64	6860.27	8945.02	< .001	0.983	0.966
Italy	colonscopia	387.020	306.701	467.338	< .001	0.954	0.911
Hungary	vastagbéltükrözés	46.545	27.729	65.360	< .001	0.854	0.729
Portugal	colonoscopia	91.741	71.137	112.345	< .001	0.953	0.908
Slovenia	kolonoskopija	41.202	24.888	57.517	< .001	0.872	0.760
Finland	kolonoskopia	29.140	25.916	32.363	< .001	0.986	0.973
Sweden	koloskopi	444.547	364.215	524.879	< .001	0.969	0.938
United Kingdom	colonoscopy	2322.180	2087.1	2557.26	< .001	0.989	0.977
Total		1482.7	1198.4	1766.8	< .001	0.961	0.923

In general, there was a significant correlation between the annual volume of performed colonoscopies and cumulative search volumes of the most related queries (R^2^ = 0.916, P<.001). Overall, a very strong correlation was observed for “colon” (r = 0.981) and “virtual colonoscopy” (r = 0.975), the strong correlation for “preparation for colonoscopy” (r = 0.721), “endoscopy” (r = 0.871), “after colonoscopy” (r = 0.819), “colonoscopy diet” (r = 0.898), and “colonoscopy under anesthesia” (r = 0.795). The weakest, moderate correlation was observed for “waiting times for colonoscopy” (r = 0.586). Apart from “colonoscopy price” (r = 0.494, P = 0.086), all correlations were statistically significant. Detailed information related to each country is presented in Table 2. 

**Table 2:** Results of univariate regression analysis between two of the most related queries search volumes and the annual number of colonoscopies performed in each of 12 included countries P<.05 was considered statistically significant

Searched term	Country	Beta - coefficient	Lower 95% CI	Upper 95% CI	P	r	R^2^
“preparation for colonoscopy”		7217.797	2614.502	11821.092	0.005	0.721	0.520
	Denmark	-506.432	-881.99	-130.86	0.013	0.689	0.474
	Germany	32745.64	22031.65	43459.7	< .001	0.907	0.823
	Ireland	560.988	14.469	1107.507	0.045	0.563	0.317
	Spain	14890.001	6747.069	23032.934	0.002	0.772	0.596
	Italy	2184.092	1314.494	3053.689	< .001	0.858	0.735
	Portugal	99.410	-6.424	205.244	0.063	0.552	0.305
	Finland	38.987	1.108	76.866	0.045	0.564	0.318
	Sweden	2491.449	224.692	4758.206	0.034	0.612	0.375
	United Kingdom	44016.7	28158.4	59875.09	< .001	0.879	0.772
“colon”		1472.246	1279.688	1664.804	< .001	0.981	0.963
	Ireland	144.613	127.252	161.973	< .001	0.984	0.968
	Spain	186.565	142.474	230.656	< .001	0.942	0.887
	France	2216.677	2097.275	2336.08	< .001	0.997	0.994
	Hungary	12.920	11.214	14.627	< .001	0.981	0.962
“endoscopy”		14302.977	8939.718	19666.236	< .001	0.871	0.758
	Denmark	29.206	-496.73	555.14	0.904	0.039	0.002
	Sweden	852.567	666.969	1038.166	< .001	0.955	0.913
“virtual colonoscopy”		43792.642	37130.399	50454.885	< .001	0.975	0.950
	Italy	2907.386	2017.893	3796.878	< .001	0.908	0.825
	Portugal	591.849	350.193	833.504	< .001	0.865	0.749
“colonoscopy price”		17481.882	-2917.782	37881.545	0.086	0.494	0.244
	Slovenia	51.233	1.264	101.201	0.045	0.586	0.343
	Finland	53.833	3.724	103.941	0.038	0.581	0.337
“after colonoscopy”		52631.9	28142.043	77121.757	< .001	0.819	0.670
	Germany	23050.298	12139.9	33960.7	< .001	0.830	0.689
	UK	24074.6	14425.36	33723.818	< .001	0.856	0.733
“colonoscopy diet”	France	145940.9	95628.5	196253.31	< .001	0.898	0.807
“colonoscopy under anesthesia”	Hungary	71.830	35.512	108.148	0.001	0.795	0.633
“waiting times for colonoscopy”	Slovenia	51.233	1.264	101.201	0.045	0.586	0.343
OVERALL		1084.9	867.16	1302.612	< .001	0.957	0.916

In the 12 analyzed countries, the weighted median age increased from 37.8 years in 2004 to 40.4 in 2015. The cumulative population increased from 350.8 to 367.6 million in the analyzed period. The number of performed colonoscopies with or without biopsies grew from 1.96 to 2.37 million, while cumulative search volume for “colonoscopy” rose from 1544 to 3015. Univariate linear regression analysis revealed that weighted median age, population, and “colonoscopy” had a very strong correlation with the number of performed colonoscopies (all P<.001, r = 0.988, r = 0.988, r = 0.961, respectively). Results are presented in Table 3.

**Table 3:** Results of univariate regression analysis between total “colonoscopy” search volumes, population, median age, and performed colonoscopies in 12 analyzed countries P<.05 was considered statistically significant

Variable	Beta coefficient	Lower 95% CI	Upper 95% CI	P	r	R^2^
“colonoscopy”	1482.639	1198.397	1766.882	< .001	0.961	0.923
median age	79207.083	71101.12	87313.05	< .001	0.988	0.977
population	0.009	0.008	0.010	< .001	0.988	0.976

## Discussion

Presented results demonstrated that overall search trends for colonoscopy increased in 12 European countries from 2004 to 2015. Data collected and published by Eurostat demonstrated a rising volume of colonoscopies with or without biopsy in Europe, during the analyzed period. Univariate linear regression analysis revealed a positive correlation between the number of performed procedures and terms “colonoscopy”, “colon”, “colonoscopy preparation”, “virtual colonoscopy”, “after colonoscopy”, “endoscopy”, “colonoscopy diet”, “colonoscopy under anesthesia”, “waiting times for colonoscopy”. These findings have multiple implications.

First, GT was validated as a useful tool in assessing public interest that may reliably estimate colonoscopy volumes. GT could be a free and easily accessible tool for institutions that oversee CRC screening programs in Europe. Balancing between screening demand and limited healthcare resources has been always a critical problem for prevention programs [25]. GT institutes a potential solution, also in accordance with other disciplines. The use of GT could be a powerful tool for predicting demand for cataract surgery, bariatric surgery, and oral and maxillofacial surgery as well [12,26,27]. Correlation coefficients varied between the countries. This could be explained by the Google Search algorithm variations [12,28].

“Colonoscopy” cumulative search volumes positively correlated with the number of performed colonoscopies as well as the weighted median age and population of the analyzed countries. Similar correlations to these definitive colonoscopy volume growth predictors proved that the Internet became an important field for screening promotion. GT could potentially monitor the effectiveness of CRC screening promotional campaigns. Google Search monitoring could ensure feedback on how certain campaigns influenced public awareness. In corresponding studies, it was revealed that GT accurately estimates public interest in prostate hyperplasia and eye thyroid disease [29,30]. Then, those findings were utilized to monitor public response to TV commercials [29,30].

Public interest in colonoscopy was focused on the colon, as a part of the intestine and colonoscopy as the procedure itself. The most popular term was “colonoscopy preparation”, found in nine out of 12 analyzed countries. Correlation with the “colonoscopy diet” was also revealed. Previous studies suggested that in 15-35% of colonoscopies, bowels were not adequately prepared [31,32]. This potentially explains the presented findings. Patients did not understand how to prepare for the colonoscopy and searched for relevant information on the Internet. The term “colon” had the highest search volume among analyzed the most related queries. Less prevalent but related to the procedure were “after colonoscopy” and “endoscopy”. This could be explained by a lack of understanding of the procedure, its course, and human anatomy. It seems reasonable that patients’ educational needs may be underestimated [12]. The Internet plays a major role in patient self-education [33]. The potential solution is to create a reliable internet-based source of information that describes the procedure, bowel preparation, and anatomy in plain language. It would benefit both patients and the health professionals. Better informed patients perceive less fear and have better bowel preparation, which makes colonoscopy easier to perform by the physician [34,35]. Plain information about the preparation for the colonoscopy, the procedure, and its rationale along with online screening promotion may be beneficial. It could be hypothesized that this information would reduce perceived psychological barriers and increase CRC screening program attendance.

Significant correlations were found between “virtual colonoscopy” search volumes and the number of performed procedures in Italy and Portugal. The term “virtual colonoscopy” refers to computed tomography (CT)-based virtual colonoscopy, also called CT colonography (CTC) [36]. This technique utilizes virtual reality techniques to navigate inside a three-dimensional patient-specific colon model reconstructed from abdominal CT images, looking for polyps [36]. Some trials indicated that CTC can be a potential screening tool to supplement colonoscopy for CRC prevention [36]. This examination requires full bowel cleansing, and colon inflation through a rectal tube similar to colonoscopy [36]. Radiation risk, the challenge in detecting small polyps, the readers’ variation, and efficiency prevented CTC from becoming a screening modality [36]. However, from the layman’s perspective, “virtual colonoscopy” could be identified with certain less invasive, less unpleasant variations of colonoscopy. This term’s prevalence could be potentially related to the public interest in less invasive CRC screening. This is in accordance with recent experts’ opinions [37]. Although colonoscopy is the gold standard in CRC screening, its troublesome bowel preparation and invasive nature deter patient participation [37]. The use of noninvasive or minimally invasive methods should be increased to improve the screening program’s attendance rate [37].

Colonoscopy is an invasive and intimate procedure. Discomfort associated with the preparation or with the procedure itself is one of the most frequently mentioned concerns [38]. While general anesthesia in screening colonoscopy is not a standard, patients seek remedies for fear and unpleasantness during the procedure [38,39]. This explains why a correlation with “colonoscopy under anesthesia” was revealed. However, it was only found in Hungary, which had a relatively low search volume, thus generalizability of this finding could not be ensured. Similarly, a correlation with “waiting times for colonoscopy” was found only in Slovenia and had a relatively small search volume. This could be linked to Slovenia’s inefficient healthcare system, that suffer significant problems in recent years [40].

This study has several limitations. First, GT data were not categorized by user intent and were deidentified. Search volumes represented potential patients as well as other internet users or care providers involved in colonoscopy. Second, the choice of searched terms could be biased. Search terms other than “colonoscopy” could generate more search volumes and lead to more diverse the most related queries. A survey of prospective patients’ search term use for colonoscopy could offer a more targeted choice of queries. Translation of analyzed terms from English to the native language and in an inverse manner could influence the results. More professional language services than Google Translate also could be beneficial.

As an aside, the annual volume of performed colonoscopies correlated with searched terms, for each country. Most of the presented correlations were at least moderately positively correlated and statistically significant. Twelve included European countries made this investigation one of the most complex and diverse among PubMed-indexed articles [12,20]. 

## Conclusions

GT could be a useful tool in assessing public interest in colonoscopy. The increasing trend in colonoscopy search volume was correlated with procedure volumes. This study presented a spectrum of public concerns related to colonoscopy. It was demonstrated that the Internet has become an important field for CRC screening promotion. To the author’s knowledge, this was the first analysis of GT data on colonoscopy focused on European countries that utilized reliable statistical data. This study highlighted the GT utility for CRC program providers.
